# A glitch in the snitch: the role of linker histone H1 in shaping the epigenome in normal and diseased cells

**DOI:** 10.1098/rsob.210124

**Published:** 2021-08-04

**Authors:** Ankita Saha, Yamini Dalal

**Affiliations:** Center for Cancer Research, National Cancer Institute, NIH, Bethesda, MD 20892, USA

**Keywords:** histone H1, chromatin, chromatin structure, epigenome

## Abstract

Histone H1s or the linker histones are a family of dynamic chromatin compacting proteins that are essential for higher-order chromatin organization. These highly positively charged proteins were previously thought to function solely as repressors of transcription. However, over the last decade, there is a growing interest in understanding this multi-protein family, finding that not all variants act as repressors. Indeed, the H1 family members appear to have distinct affinities for chromatin and may potentially affect distinct functions. This would suggest a more nuanced contribution of H1 to chromatin organization. The advent of new technologies to probe H1 dynamics *in vivo*, combined with powerful computational biology, and *in vitro* imaging tools have greatly enhanced our knowledge of the mechanisms by which H1 interacts with chromatin. This family of proteins can be metaphorically compared to the Golden Snitch from the Harry Potter series, buzzing on and off several regions of the chromatin, in combat with competing transcription factors and chromatin remodellers, thereby critical to the epigenetic endgame on short and long temporal scales in the life of the nucleus. Here, we summarize recent efforts spanning structural, computational, genomic and genetic experiments which examine the linker histone as an unseen architect of chromatin fibre in normal and diseased cells and explore unanswered fundamental questions in the field.

## Graphical abstract

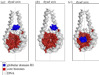

## 1. Introduction

It is a remarkable feat of engineering when approximately 3 × 10^9^ bp of DNA can be folded into ordered structures to fit inside a nucleus roughly 10^−5^ m in diameter [1]. In eukaryotes, genomic DNA is arranged inside the nucleus in a hierarchical fashion in a large nucleoprotein complex called ‘chromatin’ [2]. The word ‘chromatin’ was first introduced in 1879 by Walther Flemming as a temporary term to describe stainable material inside the nucleus to be used until its chemical nature was known [3,4]. With the rise of biochemical techniques and advances in microscopy in the late nineteenth century, the interest in the stainable substance and the morphological basis of heredity grew, leading to the isolation of ‘nuclein’ in 1869 by Friedrich Miescher. ‘Nuclein’ was later replaced with ‘nucleic acid’ as its acidic nature was described by Richard Altmann in 1889. Meanwhile, in 1884, Albrecht Kossel isolated an acid-extractable peptone-like component from goose nuclei, named it ‘histone’, and suggested that they are bound to ‘nucleic acid’ [5–8] (table 1).

**Table 1.** A summary of extant histone H1 variants across species.

	method of study	domain used for modelling	mode of binding	nucleosome-binding affinity		
				without PTM	main PTMs	proposed effect
H1.0	cryo-EM, computational modelling with crystal structures, AFM	globular domain, NTD, CTD	on-dyad	high compaction	N-terminal deacetylation and deamidation	loss of efficient binding
H1.4	computational modelling with crystal structures	globular domain, full length	off-dyad	high compaction	phosphorylation, methylation, ubiquitination	site and cell cycle-specific can lead to loss or enhanced compaction
H1.5	cryo-EM, computational modelling with crystal structures, AFM	globular domain, globular domain with truncated CTD	on-dyad	high compaction	phosphorylation, ubiquitination, acetylation	reduced affinity
H1.10	cryo-EM	full length	on-dyad	moderate to low compaction	phosphorylation, citrunillation acetylation	loss of affinity

During the subsequent century, remarkable strides were made probing the roles of histones. The structural contribution of histones towards was highlighted with the discovery of the repetitive structure of chromatin. These important insights primarily came from several nuclease digestion experiments that yielded discrete fragment sizes of 180–200 bp [9,10], which is now known to represent DNA associated with a single nucleosome. Further evidence of a structure containing distinct complexes of DNA and protein arrayed along a DNA backbone came from Woodcock (1973) and the Olinses (1974), using transmission electron microscopy to capture the first images of chromatin, the striking ‘beads on a string’ motif [11–13]. The fundamental unit of this repeating structure is the nucleosome, which is made up of approximately 147 bp of DNA wrapped around an octamer of the four core histones H2A, H2B, H3 and H4 [13]. Initially, it was thought that only a small portion of the cellular DNA is organized in nucleosomal repeats, but in 1974, Noll demonstrated by micrococcal nuclease digestion of whole nuclei that more than 80% of DNA is incorporated into nucleosomes [14], finally cementing that eukaryotes organize the majority of their genomes in a manner fundamentally distinct from the prokaryotes. Later experiments with micrococcal nuclease digestion followed by sucrose gradient fractionation, analysed by a mobility shift assay revealed complexes larger than the standard nucleosome. These complexes contained a surprise—yet another histone protein—which turned out to be even more positively charged, the tripartite linker histone or H1 [15]. Subsequent isolation of the H1 bound nucleosome demonstrated it contained ∼166 bp of DNA and was referred to as the ‘chromatosome’ [16,17]. H1 was found to bind to the DNA stretch between nucleosomes called ‘linker DNA’.

Several biophysical techniques were applied to investigate the impact of H1 on nucleosome integrity [18]. The studies showed that H1 association to the nucleosome resulted in the stability of DNA within the nucleosomal particle as well as the linker DNA [19]. This led to the suggestion that H1-induced changes contribute to the folding of nucleosomal arrays into the chromatin fibre [20–24]. In order to gain a deeper understanding of how H1 contributes to the architecture of chromatin under normal conditions versus in diseased states, it is important to understand the properties of the protein in question.

### 1.1. Linker histone H1 and its variants

The linker histone H1 protein is lysine-rich and one of the most positively charged proteins found in a cell [25]. H1s are larger than the core histone proteins with a molecular weight greater than 20 kDa. They are less tightly bound to DNA compared to the core histones and are prone to dissociation from chromatin in solutions of moderate ionic strength approximately 350 mM NaCl. The binding of the H1 protein to chromatin influences the nucleosome repeat length (NRL) and stabilizes higher-order chromatin structures. Initially, H1 was merely regarded as a structural component of chromatin; however, studies over the last two decades have demonstrated a surprisingly dynamic nature to this protein [26,27]. Like the core histone proteins, H1 also exists in variant forms [28–30].

The H1 multigene family consists of the largest number of isoforms with numerous arrangements in the genome including clustered and solitary genes [31]. *Caenorhabditis elegans* has eight variants, *Xenopus laevis* has five variants, *Gallus gallus* has seven variants, while both humans and mice have 11 [32–36]. *Drosophila melanogaster* express a single H1– dH1 in larval and adult stages [18]. However, they also express an embryonic H1 called dBigH1 before cellularization. This is replaced by dH1 when the zygotic genome is progressively activated [37,38]. There are 11 different mammalian H1 variants including seven somatic variants (H1.0–H1.5 and H1.X), three testis-specific variants (H1t, H1T2 and H1LS1) and one oocyte-specific variant (H1oo). The genes coding for H1.1–H1.5 and H1t are found clustered with the core histones on chromosome 6 (major cluster) and chromosome 3 (minor cluster). Human H1.0 is found as a single copy gene on chromosome 22 and chromosome 15 for mice [39–41]. H1s are also classified as replication-dependent (H1.1–H1.5) and replication-independent (H1.0, H1X). H1.1–H1.5 and H1X are ubiquitously expressed, whereas H1.0 is seen to accumulate in terminally differentiated cells [36]. Given the large variety of H1 proteins in existence, it is not a stretch to postulate that the differences in structure within the family would influence the outcome of its binding to the chromatin.

#### 1.1.1. H1 structure and binding dynamics

The binding affinity of the histone H1 family is dynamic. Experiments with GFP-tagged H1s demonstrate that a large fraction of nuclear H1 is bound to chromatin but continually undergoes exchange between several regions of chromatin. The binding affinity between H1 and nucleosomes determines the residence time of these molecules on the chromatin [42]. A primary determinant of this affinity is a structural component of H1—the C-terminal domain (CTD) [43]. Eukaryotic H1 has a tripartite structure containing a short N-terminal domain, a highly conserved central globular domain and long, highly disordered CTD [44]. The differences between the H1 variants are mainly found in the NTD and the CTD, thereby imparting varying binding potentials. Deletions of any of these three structural components result in reduced affinity with the loss of CTD having the strongest impact [45,46]. Fluorescence recovery after photo-bleaching (FRAP) experiments conducted on NTD green fluorescent protein-tagged H1 variants (H1.0–H1.5) showed that H1 with the shortest CTDs have the shortest residence time on nucleosomes. Furthermore, CTD swapping between H1.1, H1.4 and H1.5 and CTD truncation experiments on H1.5 reveal that the CTD is the determinant for *in vitro* binding affinity to the nucleosomes [47]. While the length of the H1 CTD plays a significant role in determining binding affinity, they are also the target for several post-translational modifications (PTMs) which may alter the availability of the CTD regions required to bind nucleosomes [48]. The effect of some of these PTMs will be discussed in a later section. Before delving deeper into the extraneous modulators of H1 binding, it is important to gain an understanding of how H1 interacts with the nucleosome because other members of the protein network like high mobility group proteins, nucleosome modifiers like the SWI/SNF complex, transcription factors and histone-modifying enzymes like histone acetyl transferases compete for the same nucleosomal binding sites. This outcome of this competition could determine the local chromatin architecture and functional outcome at a given locus.

#### 1.1.2. H1 on-dyad versus off-dyad binding

**Figure 1.**
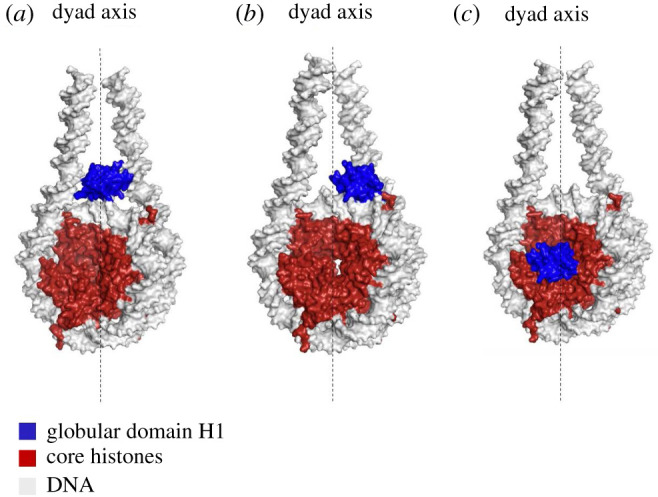
Schematic of H1-binding modes. (*a*) ‘On-dyad binding’ of the globular domain of H1 to a nucleosome. (*b*) Off-dyad binding of the globular domain H1 to a nucleosome. (*c*) ‘Dyad escaped’ H1-binding mode where H1 binds to the acidic patch of the nucleosome.

In recent years, an avenue of deep focus has been determining structural ensemble distribution of individual linker histone–nucleosome complexes. There are two widely known models of H1 binding to the nucleosome: ‘on-dyad’ or ‘off-dyad’. In the on-dyad mode, the linker histone is situated along the dyad axis as illustrated in figure 1. This localization is suggested to result in relatively poor compaction, proposing a role in transcription accessibility and more dynamic chromatin architecture. It can also give rise to alternate forms of compact chromatin which when subjected to different ionic conditions induces chromatin to unravel and reveal a ladder-like conformation [49,50]. On the other hand, the ‘off-dyad’ configuration of H1, the linker histone binds adjacent to the DNA major groove, approximately three to seven base pairs away from the dyad axis. This configuration was recently found to be critical to the formation of a spiral twist between tetranucleosomal units which in turn allows further stacking and condensing of a chromatin fibre [51,52].

Variability in H1 binding to the nucleosome can lead to different levels of mechanical stability affecting overall genomic packaging, accessibility of genomic DNA to transcriptional machinery and may even regulate nuclear processes [53–55]. For example, dH1 in both its full length and truncated forms appeared to favour off-dyad binding where the globular domain of H1 binds to only one of the linker DNA arms adjacent to the dyad axis of the nucleosome [56,57]. Full length H1.4 also appeared to bind off-dyad to a nucleosome consisting of *X. laevis* core histones. On the other hand, chicken H5 was observed to bind ‘on-dyad’ to a nucleosome containing *D. melanogaster* core histones. Full length *X. laevis* H1, truncated human H1.5 and H1.5 globular domain also bound with the globular domain ‘on-dyad’ to nucleosomes containing human core histones [49]. Another study used globular H1.0 to show on-dyad binding versus off-dyad binding of generic globular H1 to nucleosomes. The study also observed that a small number of mutations on the underlying DNA residues can shift the equilibrium of globular H1.0 binding to off-dyad, suggesting that the thermodynamic equilibrium between these two states is dependent on specific linker histone–nucleosome contact points [58].

Most of the studies above focused on the globular domains of H1 which does not take into consideration the primary determinant of H1 binding, the H1 CTD. The disordered domains are very challenging to model, but new efforts into defining the CTD for some H1 variants have been fruitful. Recent studies have showed that H1.0, H1.4 and H1.10 globular domains bind nucleosomes ‘on-dyad’ using specific residues of the NTD, the α3 helix, and L-1 loop of the globular domain. Here, the H1 CTDs play a predominant role in determining the linker DNA closeness in a nucleosome. H1.0 and H1.4 were found to simultaneously interact with both linker DNAs and bringing them closer to each other [59,60]. In a recent collaborative study from our laboratory, molecular modelling was used to investigate the effect of H1.0-disordered domains or H1 tails on chromatosome structure. Here, we reported a novel mode of H1 binding where the globular H1.0, in the absence of the ‘balancing action’ of NTD and the CTD being tethered to linker DNA, can escape the nucleosomal dyad and move towards the core histone acidic patch. This observation may represent a condition wherein PTMs on the H1 tails prevent linker DNA binding and thereby induce a ‘new’ state of H1 binding and downstream effects (figure 1*c*) [61].

#### 1.1.3. H1 and its influence on chromatin arrays

A cautionary point to consider is that the studies above were conducted using mononucleosomes, many with artificial DNA sequences. These provide powerful insights into binding probabilities, but likely do not accurately articulate the true state of chromatin fibre *in vivo*. However, powerful H1 binding to nucleosomal arrays have also been studied. For example, cryo-EM of chromatin arrays bound to H1 shows that under low salt conditions, H1 can drive the formation of a highly compact, twisted 30 nm fibre from a 12-nucleosome array. H1 shows a 1 : 1 stoichiometric association with the nucleosome cores and directly binds the nucleosome dyad and interacts with the entering and exiting linker DNA. They observe asymmetric or ‘off-dyad’ binding in tetranucleosomal units and mononucleosomes and posit that this domain localization of H1 plays a crucial role in the formation of a twist between tetranucleosomal units and thereby the 30 nm fibre [50,51]. A later study reported the crystal structure of H1-bound six-nuclesome array which was strikingly different from the compact 12-nucleosome array, to reveal a flat ladder-like conformation that was less compact. However, the authors also demonstrate that both fibre conformations can exist in a dynamic equilibrium which can be modulated by minor changes in the ionic environment [50]. It is noteworthy that H1 exists as variants in most organisms which may result in differing preferences of nucleosomal localization and elicit varying degrees and dynamism of chromatin compaction [62].

Although these conditions are not direct evidence of the chromatin state *in vivo*, the fundamental biophysical and biochemical properties driving H1 interaction with chromatin may be applicable. These principles governing H1 binding *in vitro* and *in silico* can help predict functional outcomes with regard to chromatin dynamics in diseases where H1 is altered.

### 1.2. Role of linker histones in promoting cancer

#### 1.2.1. H1 mutations in cancer

A fundamental concept that has gained traction in cancer research is that chromatin state, controlled by genetic and epigenetically alterations of histone proteins, histone-modifying enzymes and chromatin remodellers, modulates the initiation and progression of cancer. Here, we will focus on the implications of altered histone H1 which has emerged as a key player in regulating chromatin states by controlling the formation of higher-order chromatin fibres and may thereby facilitate cellular transformation.

Recent evidence of mutations in H1 variants leading to differences in H1 function has been found to have serious functional implications. For example, eight somatically acquired and seven germline mutations in *HIST1H1B-E* have been reported in follicular lymphoma (FL) [65]. The high rate of mutations observed in a cohort of 114 patients makes this gene family one of the most frequently mutated gene targets in FL. Most of the mutations were restricted to the DNA-binding CTD of the H1s with occasional indels and non-sense mutations at the globular domains. These mutated proteins were unable to bind the DNA methyltransferase DNMT1 and bound with quantitative differences with DNMT3B, suggesting an overall shift in binding affinities [64,65]. These data indicate that altering H1s may lead to changes in binding affinity with other proteins and may also interfere with the H1's ability to bind chromatin, given that the mutations were observed in the DNA-binding CTD and globular domain (GD), both of which are important for faithful H1 binding as discussed in the previous section [51].

**Figure 2.**
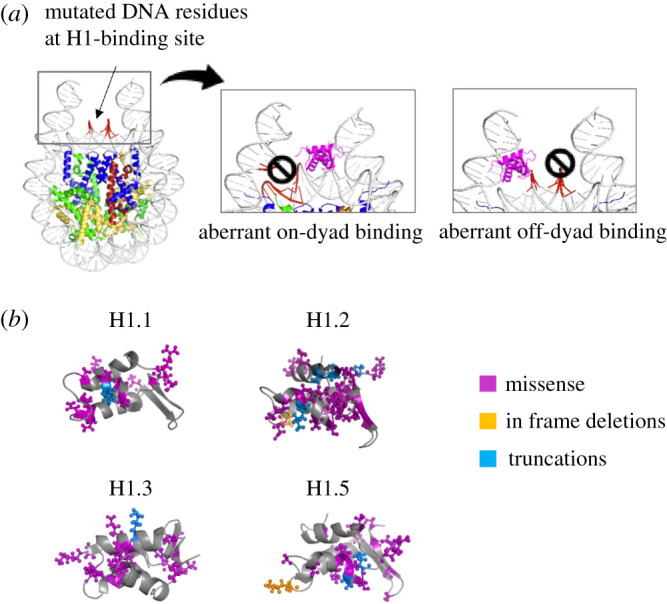
Mutations associated with cancer may affect the way H1 interacts with the nucleosome. (*a*) Mutations in the underlying residues of the DNA at the H1-binding site can push the H1 to aberrant positions. (*b*) 3D structures of select H1 variants associated with ‘driver-mutations’ in cancer. All identified mutations and the affected side chains are represented in colour.

Another set of mutations have been reported in the *HIST1H1B-E* which result in a disruption of H1 function [66]. This drives an overall shift towards a more relaxed chromatin state, downstream transformation of epigenetic states and in turn aberrant gene expression. Here, the authors reported that normal H1 binding is required to maintain nuclear compartmentalization and alterations can push cells to adopt a malignant state [67]. Recurrent H1 mutations have also been observed in diffuse large B-cell lymphoma and colorectal cancer [68–71]. But, since they had a comparatively lower rate of mutation, they were thought to be inconsequential. However, these mutations may be the difference between the location of H1 binding leading to aberrant on-dyad versus off-dyad binding (figure 2*a*). This, in turn, may give rise to less compact ‘6mer’ like zig-zag ladder-like conformation or the highly compact ‘12mer’ like conformation [51,56]. Thereby, as the previous study suggests [66,57] that an accumulation of these changes may result in direct or indirect changes in nuclear chromatin compartmentalization and architecture.

The available 3D structures of the globular domains of H1 variants and known mutations for all of these, in addition to side chains affected, are presented in figure 2*b*. These data generated from the cBio portal can be used as a tool to visualize how subtle alterations to the structure of H1 may affect its interactions with chromatin. These data raise tantalizing possibilities that are ripe for experimental exploration—for instance, would these mutations prevent the H1 from binding at all? Will the altered H1 change the conformation of the linker DNA? Much like a kink in a garden hose at one end affects the outcome at the other end, do local conformational changes translate to genomic-scale alterations?

#### 1.2.2. H1 variant protein levels in cancer

H1 stoichiometry in mammalian chromatin is found to be in a variable ratio of nearly 0.4–1 H1 per nucleosome. Changes in this ratio were found to affect the NRL where a loss of H1 increased the NRL and an excess caused a drop [72,73]. Although the exact mechanism of this relationship is yet to be defined, it is logical to speculate that altered content of H1 in nuclei may lead to a consequential change in the genome architecture at a large scale, altering long-range DNA contacts by changing three-dimensional folding and local mechanical flexibility of the fibre. This will be an exciting avenue to pursue in future studies.

Expression analyses obtained from the Cancer Genome Atlas (TCGA) reveals that H1 variants are misexpressed in a variety of cancers (table 2). Additionally, immunohistochemical analyses have also shown this to be a common theme among several cancer types with the added stipulation that the expression patterns are heterogenous displaying inter- and intra-tumour variations. H1 content can often be correlated with the pathological status of the tumours. For instance, H1.0 levels correlate with tumour differentiation status and patient survival rate [74]. H1.0 under normal conditions is seen to accumulate during terminal differentiation in numerous cell/tissue systems and said to aid the process of cellular senescence [75]. However, this accumulation action is lost in cancerous cells leading to an increased proliferative potential, de-repression of large gene domains containing oncogenic pathway triggering factors. H1.5 overexpression is found to be a marker for high-grade and metastatic prostate cancer where the benign prostate epithelium showed very low levels of H1.5, but the tumour cells exhibited a strong and homogeneous immunohistochemical staining pattern [76].

**Table 2.** A summary of mutations and expression levels of H1s in disease.

	associated cancers (TCGA)^a^	mutations		mRNA levels	PTMs associated with cancer	implication in cancer
		total	patient cohort size			
H1.0	STAD, SKCM, KIRP, HNSC, GBM, ESCA, READ, COAD, CESC, BRCA	83	558	low	unknown	increased proliferation
H1.1	unknown	88	787	unknown	unknown	unknown
H1.2	BLCA, BRCA, PAAD, UCEC	155	857	overexpressed	phosphorylation, ubiquitination	increased proliferation
H1.3	BRCA, HNSC, LUAD, SARC	124	857	overexpressed	ubiquitination	relaxed chromatin state, loss of TAD integrity
H1.4	BLCA, BRCA, COAD, COADREAD, ESCA, LUAD, PRAD, SARC, THYM	148	859	overexpressed	phosphorylation, methylation, ubiquitination	relaxed chromatin state, loss of TAD integrity
H1.5	BLCA, COAD, COADREAD, ESCA, GBMLGG, THYM, UCEC, UCS	177	842	overexpressed	phosphorylation, methylation, ubiquitination	relaxed chromatin state, loss of TAD integrity
H1.6 (testis-specific)	BRCA	77	765	low	unknown	unknown
H1.7 (testis-specific)	unknown	103	637	na	unknown	unknown
H1.8 (oocyte-specific)	unknown	69	669	na	unknown	unknown
H1.9 (testis-specific)	KIRP	na	na	na	unknown	unknown
H1.10	unknown	49	651	na	unknown	unknown

^a^Standard TCGA cancer abbreviations used: LAML, acute myeloid leukaemia; ACC, adrenocortical carcinoma; BLCA, bladder urothelial carcinoma; LGG, brain lower grade glioma; BRCA, breast invasive carcinoma; CESC, cervical squamous cell carcinoma and endocervical adenocarcinoma; CHOL, cholangiocarcinoma; LCML, chronic myelogenous leukaemia; COAD, colon adenocarcinoma; ESCA, oesophageal carcinoma; FPPP, FFPE Pilot Phase II; GBM, glioblastoma multiforme; HNSC, head and neck squamous cell carcinoma; KICH, kidney chromophobe; KIRC, kidney renal clear cell carcinoma; KIRP, kidney renal papillary cell carcinoma; LIHC, liver hepatocellular carcinoma; LUAD, lung adenocarcinoma; LUSC, lung squamous cell carcinoma; DLBC, lymphoid neoplasm diffuse large B-cell lymphoma; MESO, mesothelioma; OV, ovarian serous cystadenocarcinoma; PAAD, pancreatic adenocarcinoma; PCPG, phaeochromocytoma and paraganglioma; PRAD, prostate adenocarcinoma; READ, rectum adenocarcinoma; SARC, sarcoma; SKCM, skin cutaneous melanoma; STAD, stomach adenocarcinoma; TGCT, testicular germ cell tumours; THYM, thymoma; THCA, thyroid carcinoma; UCS, uterine carcinosarcoma; UCEC, uterine corpus endometrial carcinoma; UVM, uveal melanoma

H1 genes are under tight transcriptional control with some being regulated at the post-transcriptional and post-translational levels. Therefore, mRNA expression may not always correlate with H1 protein levels. That being said, elucidating the content of H1 variant in a cancer is key to predicting possible functional outcomes. There is now an abundance of evidence that demonstrates distinct functions of H1 variants and variable binding affinities [74–90].

**Figure 3.**
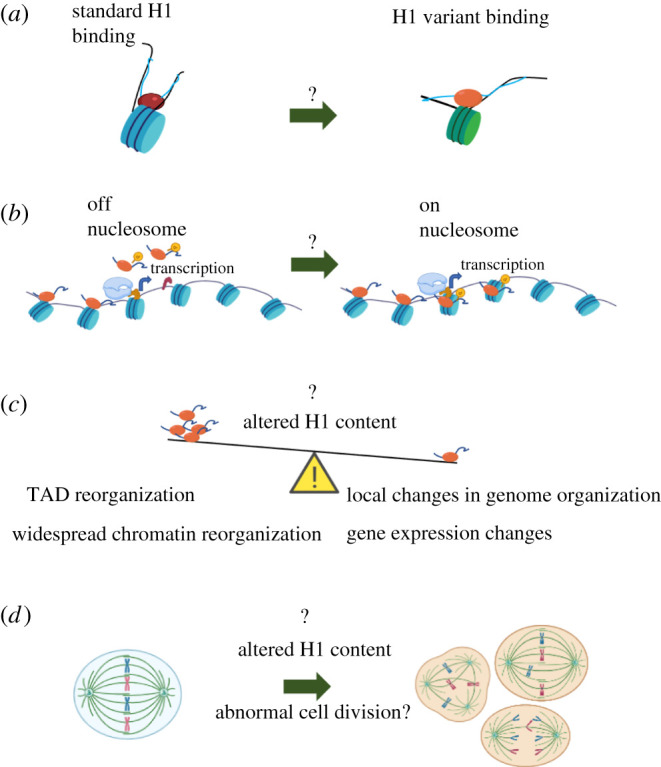
Avenues of H1 investigation that may shed light on diverse biological processes. (*a*) Modes of binding of H1 variants. (*b*) H1 variants and PTMs influence on transcription, DNA repair, etc. (*c*) H1 stoichiometry and balance influencing local and higher-order chromosome structures such as TADs; and (*d*) a role for H1 misexpression in cell division and mitotic defects in diseased cells.

The loss of protein or even the production of a mutant protein can trigger aberrant chromatin states. A recent study showed that the local density of H1 controls the balance of active or repressed domains by inducing genomic compaction and maintaining genome organizational states. A reduction in this stoichiometry can lead to a more diffuse interactive chromatin state leading to defects in cell survival pathways [77]. Another study showed that the concerted loss of H1.2 and H1.4 in breast cancer cells triggers an interferon response—a hallmark of many cancers—by de-repression of heterochromatin-associated regions [78]. Additionally, the partial redundancy among the H1 variants demonstrated using single- and compound-knockdown studies showed that when the levels of one H1 variant is reduced, the cells compensate by overexpressing other variants to maintain overall H1 levels [79,80]. However, since the different variants have varying compaction abilities, we speculate that the loss of a specific H1 variant with binding potential ‘x’ leading to a compensatory action by another H1 with binding potential ‘y’ would result in widespread chromatin architectural changes. Additionally, H1 could also bind to regions of the chromatin that it ordinarily would not, leading to changes in gene expression and chromosome architecture resulting in large-scale defects including loss of mitotic integrity (figure 3*c,d*).

#### 1.2.3. H1 PTMs in cancer

H1 variants are made up of basic amino acids with an abundance of lysine which is responsible for its highly positive electrostatic charge [47]. The lysine residue is capable of acquiring different types of PTMs, the most abundant of which is acetylation and methylation. Methylation is mostly localized to the H1 NTD, whereas acetylation is predominant in the GD [81]. Other distinct residues in H1 are modified by phosphorylation, formylation, crotonylation, ubiquitination, citrullination, 2-hydroxybutyrlation and parylation [82,83].

Phosphorylation of H1 is the most commonly found PTM and has been studied in a fair amount of depth. This PTM is particularly interesting because it appears to have a modulatory role across the cell cycle where the phosphorylation of certain H1 variants like pT146H1.4 is critical for the condensation of mitotic chromosomes and thereby appropriate cell cycle progression. Other variants, like H1X, are phosphorylated to be precluded from the mitotic chromosome.

During interphase, however, phosphorylation is associated with decondensed chromatin [84]. Some studies have shown that partial phosphorylation of H1 leads to a decreased residence time of H1 on chromatin leading to a relaxed state of chromatin [19,85], whereas others show that phospho-H1 stays associated with the chromatin even in a pro-transcriptional state [86,87].

H1 PTMs associated with cancer are the subject of extensive study in order to identify potential biomarkers for cancer progression. pT145H1.2 and pT145H1.4 have been identified as cancer biomarkers for high-grade invasive bladder cancer and metastatic progression in hepatocellular carcinoma [88,89]. Phosphorylation of H1.2, H1.3 and H1.4 at Y70 in breast cancer is positively correlated with the cell proliferative index suggesting a pro-tumour role [90]. meK84H1.4 is associated with squamous cell carcinoma of the head and neck with a poor outcome. This K84 is located in a highly conserved region of the globular domain which means that this PTM may also be seen with other H1 variants [91].

It is noteworthy that many PTMs occur in combination, primarily in the disordered regions of the H1 and some on the globular domain, which are responsible for appropriate H1 placement in a nucleosomal complex. This suggests that these PTMs can modify the manner of H1 binding and effectively modulate the chromatin state. This possibility presents an attractive challenge to structural biologists to investigate.

### 1.3. A non-nuclear, non-epigenetic, mechanical role for H1s in neurodegenerative disease

Interestingly, in addition to cancer-associated defects discussed above, there is a population of H1 predominantly found in the cytoplasm primarily associated with neurodegenerative diseases [92]. The implications and binding dynamics of non-nuclear H1 remain completely unknown and present a whole new aspect of H1 biology to explore.

This non-nuclear fraction of H1 is usually phosphorylated, most likely phospho-H1.2, and preferentially binds to the common amyloid-like structure which is made of proteins like Aβ_1–42_ and α-synuclein. These amyloid plaques are a common feature of Alzheimer's and Parkinson's diseases [93]. This study found that H1 was present in both aggregate β-amyloid plaques and diffuse plaques. They also found that H1 was enriched in the cytoplasm of neurons and astrocytes from the diseased area [94]. Another study found that H1 was upregulated in the brains of mice with symptomatic scrapie—a fatal neurodegenerative prion disease. Similar to Alzheimer's disease, increased levels of H1 were observed in the neurons and astrocytes from the diseased area of the brain [95]. While surprisingly little is known about this aspect of H1, it will be critical to the pathology and potential treatment of these diseases to elucidate, on a molecular level, how non-nuclear H1s may alter neuronal physiology and function. This kind of exploration also provides clues into the basic mechanism by which H1 might promote mechanical changes in polymers inside and outside the context of the genome.

## 2. Conclusion and future perspectives

Over the last few decades, the field of chromatin biology has made remarkable advances from first describing the ‘beads on a string’ structure to cutting edge computational modelling predicting the dynamics of chromatin in the nucleus. However, in this race to decode the details of chromatin organization, the linker histone family is now finally beginning to catch up, inevitably leading to many questions. We discuss here the various modes of H1 binding to chromatin in both mononucleosome form and arrays. But, since most of the predictions are based on the properties of globular domains, the effect of different H1 tail domains remains to be determined. How much of an effect would these different tails make? One possibility is that the addition of the H1 tail helps them bind to nucleosome conformations that are thought to preclude H1 binding due to the linker DNA proximity (figure 3*a*). Since tails are primarily the target of PTMs, these could contribute to different functional chromatin states. For example, in the pro-transcription environment, modified H1 might stay associated with the nucleosome to adjust the conformation such that it enables or guides transcription machinery towards it (figure 3*b*). Approaches that use genome-wide mapping such as CUT&RUN, or single-molecule tracking in living cells, will prove powerful allies in the quest to uncover how H1s are localized, what (if any) chromatin and DNA motif features they prefer, and the functional outcomes of their structural changes upon the nucleosome and fibre.

Initially thought to be a merely a global repressor, it is now apparent that the H1 family of proteins have the potential to affect the genome architecture through not only their intrinsic properties but also by the addition of structural conformations. It is tempting to visualize H1 dynamics in the form of a high-energy Quidditch match in a dense nuclear forest, with H1 taking the form of the ever-buzzing golden Snitch zipping in and out and around Bludger-like chromatin remodellers and Quaffle-like transcription factors, to determine the final outcome of the epigenetic game of accessibility in the eukaryotic nucleus.

## Data accessibility

This article does not contain any additional data.
